# Room temperature polaritonic soft-spin XY Hamiltonian in organic–inorganic halide perovskites

**DOI:** 10.1515/nanoph-2023-0818

**Published:** 2024-02-15

**Authors:** Kai Peng, Wei Li, Natalia G. Berloff, Xiang Zhang, Wei Bao

**Affiliations:** Department of Materials Science and Engineering, Rensselaer Polytechnic Institute, Troy, NY, USA; Nanoscale Science and Engineering Center, University of California, Berkeley, CA, USA; Department of Applied Mathematics and Theoretical Physics, University of Cambridge, Cambridge, UK; Faculty of Science and Faculty of Engineering, The University of Hong Kong, Hong Kong, China; Department of Electrical and Computer Engineering, University of Nebraska-Lincoln, Lincoln, NE, USA

**Keywords:** BEC, XY Hamiltonian, halide perovskites, polaritons

## Abstract

Exciton–polariton condensates, due to their nonlinear and coherent characteristics, have been employed to construct spin Hamiltonian lattices for potentially studying spin glass, critical dephasing, and even solving optimization problems. Here, we report the room-temperature polariton condensation and polaritonic soft-spin XY Hamiltonian lattices in an organic–inorganic halide perovskite microcavity. This is achieved through the direct integration of high-quality single-crystal samples within the cavity. The ferromagnetic and antiferromagnetic couplings in both one- and two-dimensional condensate lattices have been observed clearly. Our work shows a nonlinear organic–inorganic hybrid perovskite platform for future investigations as polariton simulators.

## 1 Introduction

Exciton–polaritons are quasiparticles that arise from the strong coupling between excitons in semiconductors and photons in microcavities. Due to their small effective mass inherited from photons and strong nonlinearity inherited from excitons, exciton–polaritons have been utilized to achieve Bose–Einstein condensation (BEC) at very high temperatures than cold atoms [1], [2], [3]. The traditional molecular beam epitaxy (MBE) GaAs quantum well microcavity has been used as a polariton platform to demonstrate the properties of the “quantum fluid,” such as superfluidity [4], dark soliton [5], and simulating XY Hamiltonians [6]. However, due to the small binding energy of excitons in GaAs, these experiments must be performed at low temperatures, limiting their applicability as real-life devices.

Semiconductor lead halide perovskites, as a new room-temperature polariton platform, have been researched. Because of the large Wannier–Mott exciton binding energy [7], [8], high photoluminescence (PL) efficiency [9], and large nonlinearity [10], halide perovskites have shown polariton condensation [11], polariton lattice [12], superfluidity [13], and the large-scale XY spin Hamiltonian [14] all at room temperature. However, these experiments were all performed with inorganic perovskites. As for the most mature organic–inorganic halide perovskite materials, such as MAPbBr_3_ and FAPbBr_3_, used in the research for the next generation of solar cells [15], to the best of our knowledge, room-temperature polariton condensation has never been achieved. So far, the polariton condensation has only been achieved at cryogenic temperatures in two-dimensional hybrid perovskites [16]. This is partly because of the intrinsic instability of the materials, and the limitations in growth methods and fabrication processes, as the polariton condensation experiments generally require the integration of high-quality crystals and optical microcavities.

In this work, we realized room-temperature polariton condensation with organic–inorganic halide perovskite MAPbBr_3_. Large and uniform single-crystal perovskites were grown directly in prebonded microcavities by solution method, as shown in our previous work [14]. These high-quality hybrid perovskites allowed us to attain room-temperature polariton condensation and subsequently build two-dimensional condensate lattices for simulating the soft-spin XY Hamiltonian for the first time with these materials. Our work realizes an organic–inorganic hybrid perovskite platform for room-temperature nonlinear polaritonics.

## 2 Results and discussions

### 2.1 Characterization of polaritons in MAPbBr_3_

**Figure 1:**
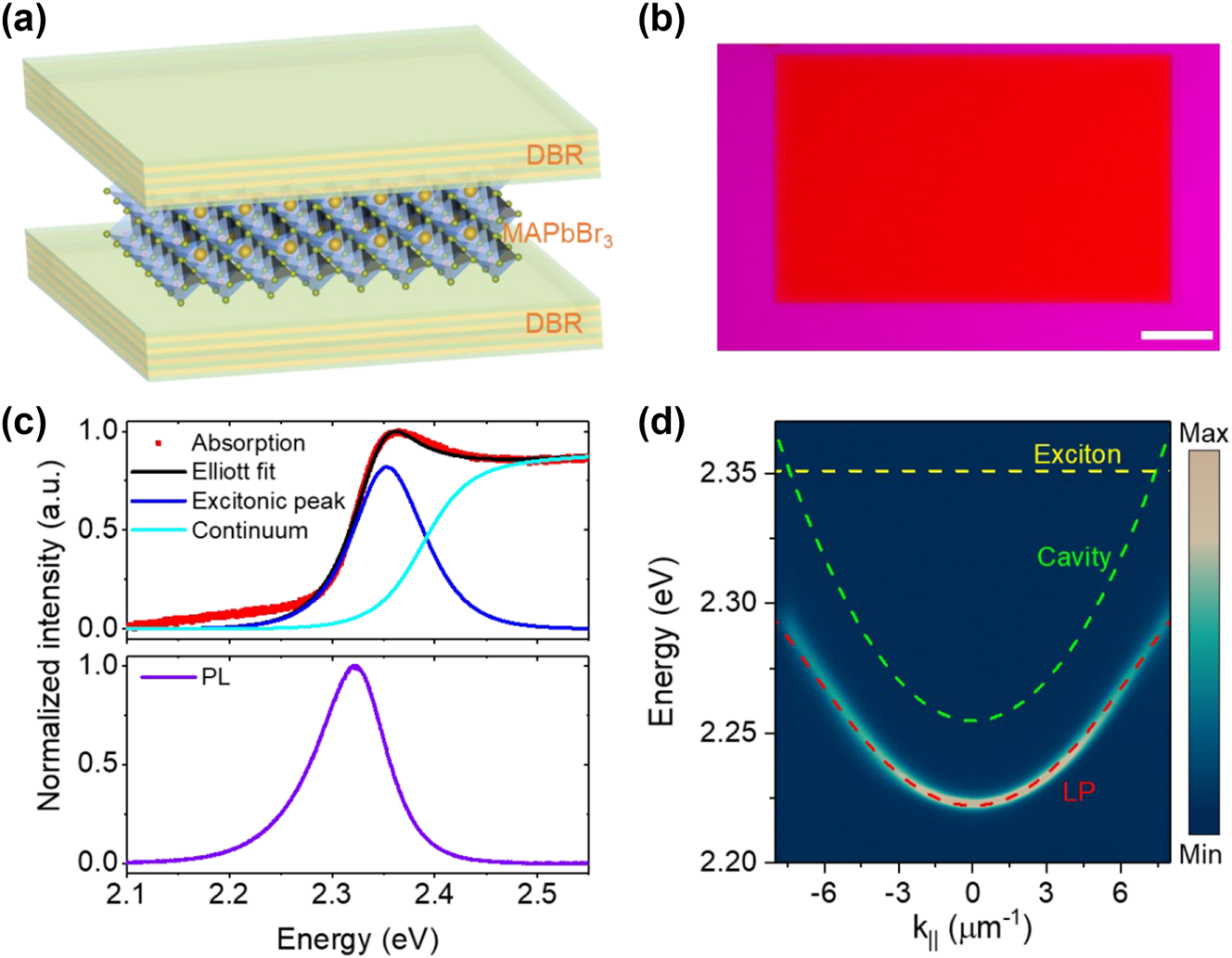
Characterization of exciton–polaritons in organic–inorganic halide perovskite microcavity. (a) Schematic of the perovskite microcavity, consisting of a solution-grown MAPbBr_3_ single crystal sandwiched between two 12.5 pairs DBR mirrors with cavity quality factor Q ∼700–800. (b) Optical image of a MAPbBr_3_ single crystal in the cavity with a transmission illumination. Scale bar: 50 μm. (c) The normalized absorption and photoluminescence (PL) spectra of a ∼200-nm thick MAPbBr_3_ single-crystal at room temperature. The absorption spectrum in the top panel was fitted by the Elliott model [20], [21]. An excitonic peak (blue solid line) and a continuum absorption curve (cyan solid line) can be observed clearly. An exciton binding energy of ∼38 meV was extracted. (d) Angle-resolved PL dispersion of the strong-coupled exciton–polariton of the perovskite cavity. The dashed lines are fitted curves of the exciton energy (exciton), cavity mode (cavity), and lower polariton mode (LP).

The cavity structure is shown schematically in Figure 1(a). The planar Fabry–Pérot cavity was made by a wafer-bond process of two DBR mirrors. Then, the single-crystal MAPbBr_3_ was grown by a solution method under the confinement of the microcavity (∼330 nm). With this method, high-quality, large-sized perovskite single crystals can be grown in the cavity, as shown in Figure 1(b). More details of the fabrications, growth, and characterizations can be found in Methods and our previous work [14]. Figure 1(c) shows the absorption and the PL spectra of the MAPbBr_3_ plate grown in the 200-nm thick empty bonded quartz substrates. Previous studies show that excitons in MAPbBr_3_ perovskite have an exciton binding energy of about 25–42 meV at room temperature [17], [18], [19], supporting room-temperature excitons. This is consistent with our fitted exciton binding energy of ∼38 meV from the absorption spectrum based on the Elliott model [20], [21] in Figure 1(c).

For the perovskite sample in the cavity, a sharp polariton dispersion can be observed via the angle-resolved PL spectrum shown in Figure 1(d). The polaritons were formed from the strong coupling between the exciton in perovskite and the cavity photons, which can be described very well with a coupled oscillator model neglecting linewidth:

(1) $$\begin{array}{c} \left(\begin{array}{cc} E_{\mathrm{cav}}\left(k_{\|}\right) & \Omega /2\\ \Omega /2 & E_{ex} \end{array}\right)\left(\begin{array}{c} a\\ b \end{array}\right)=E\left(\begin{array}{c} a\\ b \end{array}\right) \end{array}$$

Here, the cavity mode is represented as $E_{\mathrm{cav}}\left(k_{\|}\right)=E_{0}+\frac{\hbar^{2}k_{\|}^{2}}{2m}$ with mode energy *E*_0_ at *k*_‖_ = 0 and effective mass *m* of the cavity photon. *E*_*ex*_ is the exciton energy that can be extracted from the absorption spectrum in Figure 1(c). *E* is the eigenvalue of the polaritons, and *a* and *b* represent the Hopfield coefficients. From the fitting results as the dashed lines in Figure 1(d), we extracted the Rabi splitting Ω = 130 meV and a negative exciton–photon detuning of Δ = −96 meV for this sample.

### 2.2 Room-temperature polariton condensation

**Figure 2:**
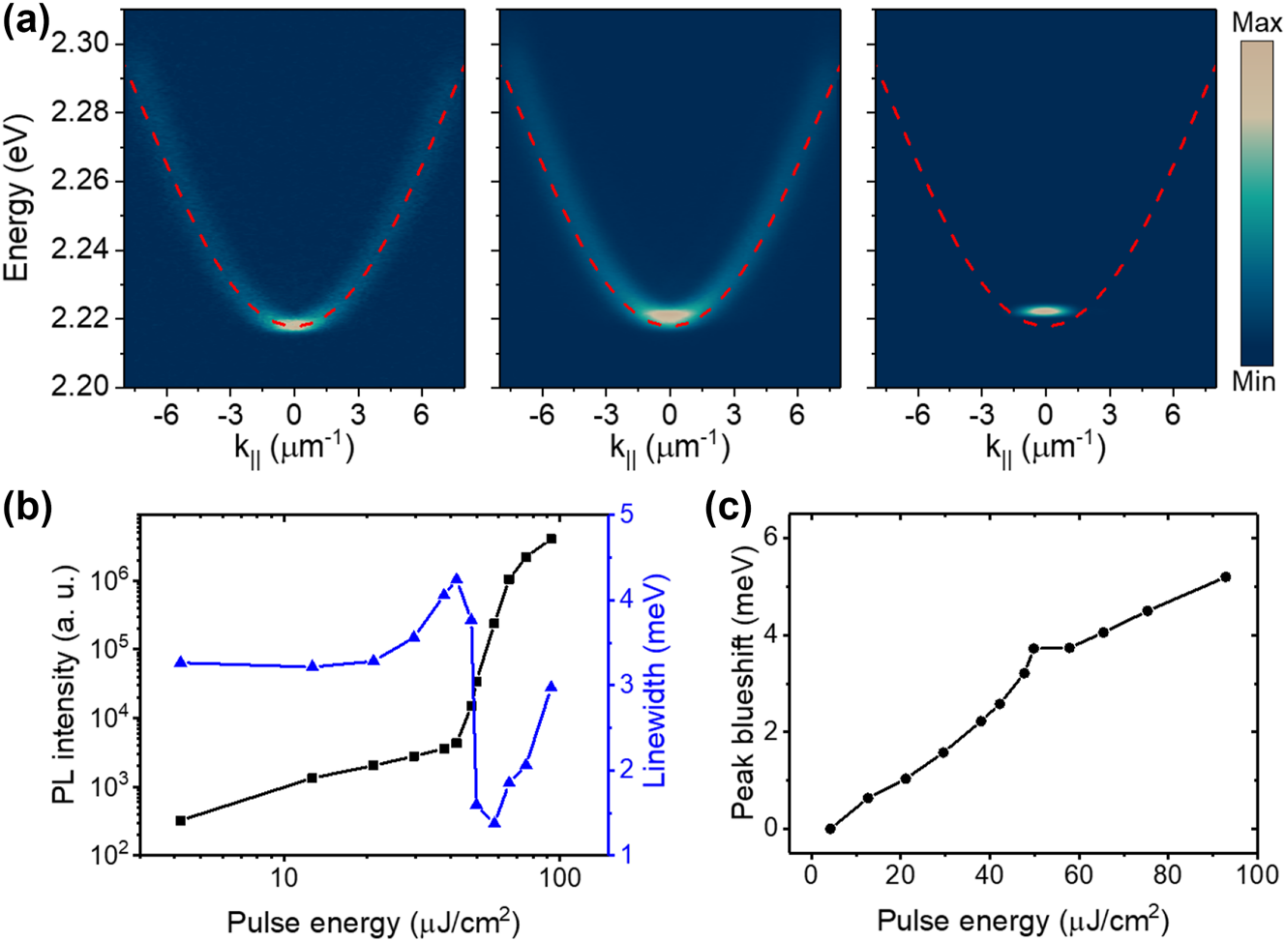
Exciton–polariton condensation at room temperature. (a) Angle-resolved power-dependent PL dispersions under nonresonant excitation at 0.1 *P*_th_, *P*_th_, and 1.5 *P*_th_ from left to right, where *P*_th_ (∼42.2 μJ cm^−2^) is the pumping power of condensation threshold. With the power increasing, the polaritons condensate at the ground state of the polariton mode. The dashed red lines are the fitted curve of the lower polariton mode with low pumping power. (b) Log–log plot of the integrated PL intensities of the ground polariton mode at *k*_||_ = 0 and the full width at half maximum versus pulse energy. The nonlinear increase of the intensity and the narrowing of the linewidth clearly demonstrate the polariton condensation. (c) PL peak blueshifts of the mode at *k*_||_ = 0. Due to the strong exciton reservoir interactions, a stronger blueshift was observed below the threshold.

The strong nonlinear properties of MAPbBr_3_ enable us to achieve polariton condensation. A 450-nm, 250-fs pulsed laser was used to excite the sample nonresonantly with a laser spot size of ∼28 μm. Figure 2(a) shows the angle-resolved power-dependent PL dispersions with pulse energy at 0.1 *P*_th_, *P*_th_, and 1.5 *P*_th_ from left to right, where *P*_th_ is the threshold of ∼42.2 μJ cm^−2^. A pinhole at the real-space imaging plane was used to only extract the PL from the condensation area. Above the threshold, the polariton ground state at *k*_||_ = 0 exhibits a strong nonlinear emission enhancement and linewidth narrowing, clearly evidences of the condensation. This is also illustrated in Figure 2(b) through the PL intensity and linewidth as a function of the pulse energy. Meanwhile, due to the polariton–polariton interactions, polariton dispersion has a continuous blueshift with the increase of density. A stronger blueshift was observed below the threshold due to the strong exciton reservoir interactions. All these typical features demonstrated the occurrence of polariton condensation in our perovskite system.

### 2.3 Soft-spin XY Hamiltonian by polariton condensate lattices

**Figure 3:**
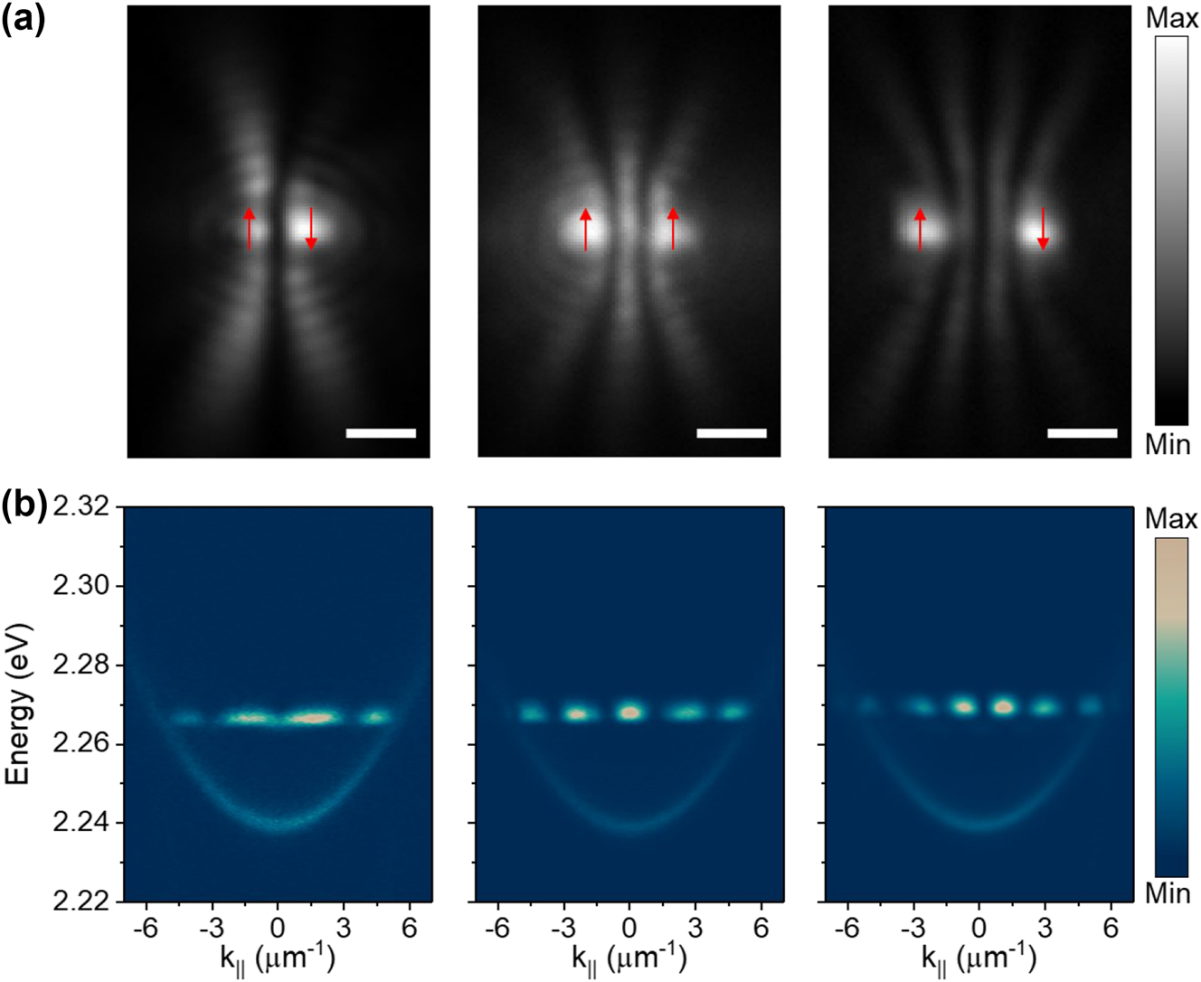
Phase configuration between two polariton condensates. (a) The time-integrated real-space images of the two polariton condensates with different separation distances of 1.78, 2.5, and 3.55 μm from left to right, respectively. With the separation distances increase, the interference fringes increase from zero to three, demonstrating the transitions from antiphase to the in-phase and back to the antiphase synchronization between the two condensates. The red arrows marked at the condensates represent the relative spin directions. Scale bars: 2 μm. (b) The corresponding angle-resolved PL dispersions of the condensates in (a). The detuning of this sample is smaller than that shown in Figure 2. The interference fringes at high energy state also demonstrate the different phase synchronizations between the two condensates.

By utilizing the long-range coherence of the condensates, we can create multiple condensates and study their interactions. Due to the repulsion interaction between polaritons, condensation can occur at a high energy state with a nonzero in-plane momentum when the pumping laser spot is small [22], [23]. Interference and phase difference synchronization occur when different out-flowing condensates meet according to previous theoretical models and the experimental results [6], [23], [24]. A polariton dyad was used to demonstrate this phase synchronization in our experiment. A phase-only reflective spatial light modulator was used to generate the designed condensate array, and more details of the experimental setup can be found in Methods. As shown in Figure 3, with the separation distances between the two condensates increasing, the interference fringes increase from zero to three, demonstrating the transitions from antiphase to the in-phase and back to the antiphase synchronization between the two condensates. The red arrows marked at the condensates represent the relative phase directions. The corresponding PL dispersions in Figure 3(b) also demonstrate these different phase synchronizations.

**Figure 4:**
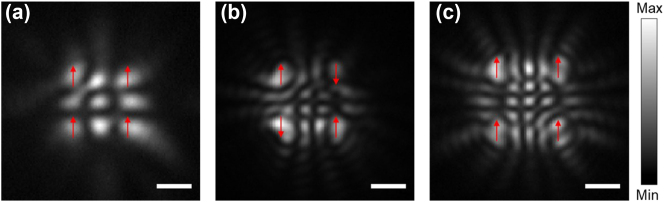
Construction of the two-dimensional polariton spin lattices. (a–c) The time-integrated real-space images of the 2 × 2 polariton condensate square lattices. With increasing lattice distance, the condensate lattices undergo a transition from ferromagnetic to antiferromagnetic and then to the next ferromagnetic coupling states in (a)–(c), respectively. The red arrows marked at the condensates represent the relative spin directions. Scale bars in (a)–(c): 2 μm.

To validate the capability of our sample to construct a large-scale polariton condensate lattice, we assembled a 2 × 2 square lattice to simulate the two-dimensional soft-spin XY Hamiltonian. As shown in Figure 4(a), one interference fringe can be observed between the nearest condensates, demonstrating the in-phase coupling and the ferromagnetic states. Like the one-dimensional cases in Figure 3, with the separation distance increase between the condensates, antiferromagnetic and the next ferromagnetic coupling states can be obtained from the even and odd interference fringes between the nearest condensates in Figure 4(b) and (c), respectively.

### 2.4 Theoretical background

We model the lattice of polariton condensates as the system of coupled oscillators [25]

(2) $$\begin{array}{ll} \frac{\text{d}\Psi_{i}}{\text{d}t} & =\left(\gamma^{\mathrm{inj}}\left(t\right)-\gamma_{c}-{\left|\Psi_{i}\right|}^{2}\right)\Psi_{i}+\underset{j,j\neq i}{\sum }J_{ij}\Psi_{j}\\ & \;-iU{\left|\Psi_{i}\right|}^{2}\Psi_{i}, \end{array}$$

where $\Psi_{i}=\sqrt{\rho_{i}}\exp\left[i\theta_{i}\right]$ is the complex amplitude of the *i*th oscillator, *ρ*_*i*_ (*θ*_*i*_) is its number density (phase), $\gamma^{\mathrm{inj}}\left(t\right)$ is the incoherent injection/gain (assumed uniform for all oscillators), *γ*_*c*_ represents the linear losses, *U* is polariton–polariton interaction strength, and *J*_*ij*_ is the coupling strength between *i*th and *j*th oscillator. We write Eq. (2) as dimensionless; the exact nondimensionalization as well as the approximations used in deriving Eq. (2) can be found in [25]. The coupling strength depends on the number of parameters: shape of the excitation/injection, its intensity, and the distance *d*_*ij*_ between the pumping sites [26]. The first two parameters determine the in-plane momentum of the condensates *k*_*c*_ and the coupling strength varies as a Bessel function of the first kind $J_{ij}\approx J_{0}\left(k_{c}d_{ij}\right)$ taking positive (ferromagnetic coupling) and negative (antiferromagnetic coupling) values [23], [26], [27]. We demonstrate this experimentally by increasing the separation between two condensates (a polariton dyad [28]) and observing the ferromagnetic (in-phase) and antiferromagnetic (antiphase) couplings shown in Figure 3.

The system of Eq. (2) is obtained from the mean-field Ginzburg–Landau equations [29] governing the evolution of the condensates in the lattice using tight-binding approximation integrating over spatial degrees of freedom [25]. The real terms of this equation represent the gradient descent of the loss function *F*_*loss*_ for *N* condensates:

(3) $$\begin{array}{ll} F_{\mathrm{loss}} & =\overset{N}{\underset{i=1}{\sum }}\left(\frac{1}{2}{\left|\Psi_{i}\right|}^{4}-\left(\gamma^{\mathrm{inj}}\left(t\right)-\gamma_{c}\right){\left|\Psi_{i}\right|}^{2}\right)\\ & \;-\frac{1}{2}\underset{i,j}{\sum }J_{ij}\left(\Psi_{i}^{*}\Psi_{j}+\Psi_{i}\Psi_{j}^{*}\right) \end{array}$$

as

(4) $$\begin{array}{c} \frac{d\Psi_{i}}{dt}=-\frac{\delta F_{\mathrm{loss}}}{\delta \Psi_{i}^{*}} \end{array}$$

while the last conservative term of Eq. (2) contributes to the phase rotation. The oscillator system minimizes the system’s losses via graduate descent while the injection rate *γ* ^inj^ acts as the bifurcation (annealing) parameter, taking the system through the Andronov–Hopf bifurcation [30]. The operation of the system can be compared and contrasted with the Coherent Ising Machine [31], [32], [33], [34], [35], [36], [37], which is the real-valued analog of Eq. (2) where the phases are restricted to take values *θ*_*i*_ ∈ {0, *π*} and *U* = 0. When the injection rate (laser power) is small, so $\gamma^{\mathrm{inj}}\left(t\right)-\gamma_{c}<0$ the loss function landscape is convex, with the only minimum occurring at Ψ_*i*_ = 0 for all *i*. As $\gamma^{\mathrm{inj}}\left(t\right)$ increases, the loss landscape will become nonconvex when the complex Hessian matrix with components $H{\left(\Psi \right)}_{ij}=\frac{\delta^{2}F_{\mathrm{loss}}}{\delta \Psi_{j}\delta \Psi_{i}^{*}}$ acquires a negative eigenvalue. We can bound the minimum eigenvalue $\lambda_{\min}\left(H\left(\Psi \right)\right)$ of *H*, by Jensen’s inequality

(5) $$\begin{array}{c} \lambda_{\min}\left(H\left(\Psi \right)\right)\geq 2\underset{i}{\min}{\left|\Psi_{i}\right|}^{2}-\gamma^{\mathrm{inj}}+\gamma_{c}-\lambda_{\max}\left(J\right). \end{array}$$

Therefore, $\lambda_{\min}\left(H\left(\Psi \right)\right)<0$ (and *F*_*loss*_ can become non-convex) when $\gamma^{\mathrm{inj}}>\gamma_{c}-\lambda_{\max}\left(J\right)\equiv \Gamma \left(J\right)$ . As *γ* ^inj^ increases, the loss landscape becomes nonconvex and new minima appear along the direction defined by the eigenvector associated with the largest eigenvalue of the coupling matrix *J*. As the injection increases further, the minima deviate from those defined by the spectral (linear) problem toward the true global minimum of the loss function. With the further injection increase $\gamma^{\mathrm{inj}}\left(t\right)\gg \Gamma \left(J\right)$ , the densities tend to minimize the first term of Eq. (3) and reach *ρ*_*i*_ = *γ* ^inj^ − *γ*_*c*_ to the leading order while the phases minimize the classical XY Hamiltonian $H_{XY}=-\underset{i,j}{\sum }J_{ij}\cos\left(\theta_{i}-\theta_{j}\right)$ . However, in this case, minimization of *H*_*XY*_ becomes a weak perturbation of the uncoupled system of oscillators. In an analogy with the soft-spin Ising Hamiltonian [38], we refer to *F*_*loss*_ defined in Eq. (3) as “soft-spin” and to *H*_*XY*_ as “hard-spin” XY Hamiltonians.

Finally, more information about the dynamics can be gathered by separating real and imaginary parts of Eq. (2):

(6) $$\begin{array}{c} \frac{1}{2}{\dot{\rho }}_{i}=\left(\gamma^{\text{inj}}\left(t\right)-\gamma_{c}-\rho_{i}\right)\rho_{i}+\underset{j,j\neq i}{\sum }J_{ij}\sqrt{\rho_{j}\rho_{i}}\cos\left(\theta_{i}-\theta_{j}\right) \end{array}$$

(7) $$\begin{array}{c} {\dot{\theta }}_{i}=-U\rho_{i}-\underset{j,j\neq i}{\sum }J_{ij}\sqrt{\frac{\rho_{j}}{\rho_{i}}}\sin\left(\theta_{i}-\theta_{j}\right) \end{array}$$

The fixed point of Eq. (6) at a particular saturation value of *γ* ^inj^ yields

(8) $$\begin{array}{c} \rho_{i}=\gamma^{\mathrm{inj}}-\gamma_{c}+\underset{j,j\neq i}{\sum }J_{ij}\sqrt{\frac{\rho_{j}}{\rho_{i}}}\cos\left(\theta_{i}-\theta_{j}\right) \end{array}$$

while Eq. (7) represents the Kuramoto oscillators with self-frequencies *Uρ*_*i*_; however, the coupling strengths between the Kuramoto oscillators are modified by their amplitudes. A constant relative phase between them will be achieved only if amplitudes are not vastly different.

To summarize, our analysis indicates that as *γ* ^inj^ increases, the system undergoes several transitions and for each fixed value of *γ* ^inj^ solves a different problem. Close to the condensation threshold when $\gamma^{\mathrm{inj}}\approx \Gamma \left(J\right)$ , the system evolves from Ψ_*i*_ = 0 state into the state along the negative curvature direction dictated by the principle eigenvector of the coupling matrix *J*; however, it stabilizes to a fixed point in relative phases only at a higher *γ* ^inj^ when amplitudes are sufficiently homogeneous. This fixed point corresponds to the minimum of the loss function, which is a soft-spin version of the classical XY Hamiltonian. In the high injection limit, $\gamma^{\mathrm{inj}}\gg \Gamma \left(J\right)$ , the spin densities approach *γ* ^inj^ − *γ*_*c*_ with the relative phases reaching a fixed point corresponding to the minimum of the hard-spin XY Hamiltonian. However, the minimization comes as a perturbation. In our experiments, therefore, we do not reach the high intensity limit and observe the minimization of the soft-spin XY Hamiltonian represented by the loss function given by Eq. (3). The density heterogeneities are clearly observed on Figure 4.

**Figure 5:**
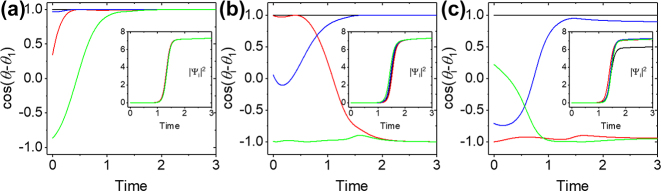
The time evolution of the phases and the squares of the complex amplitudes of four condensates. (a–c) The phases of the condensates represented by $\cos\left(\theta_{i}-\theta_{1}\right)$ obtained by numerical integration of the system of Eq. (2) starting with random initial conditions with *J*_12_ = *J*_23_ = *J*_34_ = *J*_14_ = 1 (a) and *J*_12_ = *J*_23_ = *J*_34_ = *J*_14_ = −1 (b, c) interactions along the sides of the square formed by four condensates. Here $U=1,\gamma^{\mathrm{inj}}\left(t\right)=10\tanh\left(t/100\right)+10,\gamma_{c}=5$ . The insets depict the squares of the complex amplitudes ${\left|\Psi_{\text{i}}\right|}^{2}$ . The solid lines in black, red, blue, and green represent the phase and the square of the amplitude of the condensates with *i* = 1 ∼ 4, respectively. (c) The same as (b) but the linear losses are *γ*_*c*1_ = 1.2*γ*_*ci*_, *i* ≠ 1. The single shot experiment phase deviations discussed in the main text are *δθ*_1_ = ±0.110*π*, *δθ*_2_ = ±0.154*π*.

To illustrate the evolution and the steady state of our system, we solved the system of Eq. (2) starting with random initial conditions with *J*_12_ = *J*_23_ = *J*_34_ = *J*_14_ = 1 ferromagnetic (F) and *J*_12_ = *J*_23_ = *J*_34_ = *J*_14_ = −1 antiferromagnetic (AF) interactions along the sides of the square formed by four condensates. Without loss of generality, we took *U* = 1, $\gamma^{\mathrm{inj}}\left(t\right)=10\tanh\left(t/100\right)+10$ , *γ*_*c*_ = 5. Figure 5 depicts the time evolution of the phases via $\cos\left(\theta_{i}-\theta_{1}\right)$ (main panels) and the squares of the complex amplitudes ${\left|\Psi_{\text{i}}\right|}^{2}$ (insets) for F (Figure 5(a)) and AF (Figure 5(b)) interactions. As the amplitudes bifurcate from the vacuum states, the phases take the values that minimize the XY Hamiltonian: {0, 0, 0, 0} for F and {0, *π*, 0, *π*} for AF interactions. For such simple configuration, the minima of the soft-spin and hard-spin Hamiltonians coincide since the amplitudes take the same values for large times. To see the realization of the soft-spin XY Hamiltonian, we will assume that one of the oscillators is located near a defect and the linear losses for that condensate differ from the rest of the system. Without loss of generality, we will assume that the linear losses are 20 % higher for that condensate, say the condensate Ψ_1_. Figure 5(c) shows the time evolution of the phases and the densities of the condensates for AF interactions. The density of the Ψ_1_ condensate is depleted with respect to the densities of the other condensates, while the phases deviate from {0, *π*, 0, *π*} by *δθ*_1_ and *δθ*_2_ and become {0, *π* + *δθ*_1_, *δθ*_2_, *π* + *δθ*_1_}, as seen in Figure 5(c). However, configurations with ± *δθ*_1_ and ± *δθ*_2_ are equally likely to be realized, so on the time-averaged experiments, like ours, the state {0, *π*, 0, *π*} is seen.

## 3 Conclusions

In summary, we realized room temperature polariton condensation and an analog simulation of a two-dimensional soft-spin XY spin Hamiltonian by polariton condensate lattices with high-quality organic–inorganic halide perovskite single crystals for the first time. Due to the higher solubility of the precursors, the resulting single-crystal hybrid perovskites tend to be much larger in lateral sizes compared to CsPbBr_3_. On the other hand, due to the relatively higher condensation threshold and vulnerability to nanofabrication compared to inorganic perovskites, this real-time tuning of the pumping laser method is well-suited for establishing condensate lattices in organic perovskite materials. As a new nonlinear room-temperature system, our cavity can be used to realize large-scale soft-spin XY Hamiltonians [6], [14] and neural networks [39], [40] for optimization problems [41], [42] by combining other algorithms and modulation techniques [43], [44], [45], [46], [47] in the future.

## 4 Methods

### 4.1 Cavity fabrication

 12.5 pairs of SiO_2_/Ta_2_O_5_ DBR mirror were deposited on six-inch well-clean quartz wafers by electron beam evaporation with an advanced plasma source. A gold pad array of 250 μm side length was deposited on the DBR surface by electron beam evaporation. Then, two of the same DBR wafers were aligned by the gold pads and bonded together in a wafer bonder. The bonded wafers were diced into 2-cm chips. More details can be found in our previous work [14].

### 4.2 Synthesis of halide perovskites

The perovskites were grown by an inverse-temperature crystallization method [48]. A droplet of precursors (1.6 M 1:1 mixture of MABr and PbBr_2_ in *N*, *N*-dimethylformamide) was deposited at the edge of the microcavity and filled the entire microcavity through capillary action. Subsequently, the crystals gradually crystallized as the solubility decreased at 80 °C for 24–48 h in a nitrogen-filled glove box. This solution growth method under confinement can yield high-quality, large-sized perovskite single crystals. After the crystal growth, the cavity with crystals was put in a vacuum chamber at an 80 °C hotplate to remove the residual solvent.

### 4.3 Optical measurements of polariton

A home-built optical setup in a transmission configuration was used for all the optical measurements. A 450-nm, 250-fs optical parametric amplifier pulsed laser with a 2 kHz repetition rate was used to pump the sample nonresonantly. A phase-only reflective liquid-crystal spatial light modulator was used to generate pumping laser patterns by holograms computed by a Gerchberg–Saxton algorithm. The laser pattern was transferred to an objective (Nikon 40× Plan Fluor ELWD, N.A. = 0.6) with a Fourier imaging configuration. The PL was collected in a transmission configuration with another objective (Nikon 40/60× Plan Fluor ELWD, N.A. = 0.6/0.7). The angle-resolved dispersions were measured by a Fourier imaging system with two achromatic tube lenses and an Andor spectrometer equipped with a two-dimensional CCD. The real-space images were obtained by a two-dimensional Princeton instrument EMCCD.
